# The Gay Parental Turn: Canadian Gay Fathers and the Reorganization of Care and Community

**DOI:** 10.1111/cars.70035

**Published:** 2026-04-06

**Authors:** S. W. Underwood

**Affiliations:** ^1^ Department of Sociology & Anthropology Simon Fraser University Burnaby Canada

**Keywords:** community, family, fatherhood, gay fathers, LGBTQ+

## Abstract

Community has long been key to the well‐being of gay men, yet little research examines how gay fathers, specifically, relate to the broader LGBTQ+ community. Drawing on interviews with 18 mostly White, (upper‐) middle‐class Canadian gay fathers, I investigate their expectations and experiences of family and community, showing they describe ‘gay community’ in terms of gay ancestors, friends, and shared gay activities. Fathers express deep gratitude for past LGBTQ+ people whose interdependent collective struggle secured their rights to marry and parent. They hope for support from family and gay friends and strive to maintain community ties, but find their efforts complicated by the demands of intensive parenting, geographic distance, and limited childcare support. As children grow, fathers build community with the straight parents of their children's friends, demonstrating how social class, whiteness, and homonormativity shape the reorganisation of these Canadian gay fathers’ lives—a process I call the gay parental turn.

## Résumé

La communauté joue depuis longtemps un rôle essentiel dans le bien‐être des hommes gays, mais peu de recherches se sont penchées sur la manière dont les pères gays, en particulier, s’inscrivent dans la communauté LGBTQ+ au sens large. En m’appuyant sur des entretiens menés auprès de 18 pères gays canadiens, pour la plupart blancs et issus de la classe moyenne (supérieure), j’étudie leurs attentes et leurs expériences en matière de famille et de communauté, montrant qu’ils décrivent la « communauté gay » en termes d’ancêtres gays, d’amis et d’activités gays partagées. Les pères expriment une profonde gratitude envers les personnes LGBTQ+ des générations passées, dont la lutte collective et solidaire a permis de garantir leurs droits au mariage et à la parentalité. Ils espèrent bénéficier du soutien de leur famille et de leurs amis gays et s’efforcent de maintenir des liens communautaires, mais leurs efforts sont compliqués par les exigences d’une parentalité intensive, la distance géographique et le manque de soutien en matière de garde d’enfants. À mesure que les enfants grandissent, les pères tissent des liens avec les parents hétérosexuels des amis de leurs enfants, démontrant ainsi comment la classe sociale, la blancheur et l’homonormativité façonnent la réorganisation de la vie de ces pères homosexuels canadiens — un processus que j’appelle le « tournant parental gay ».

## 1 Introduction

In the late 20th century, gay community was a key source of friendship and family, providing the material and social support needed to sustain gay family life (Weston 1991; Weeks et al. 2001). Long before legal rights—especially during the AIDS epidemic—being “in community” was a vital survival strategy. Indeed, LGBTQ+ people are often assumed to access ready‐made communities offering belonging, identity, and support (Formby 2017a, 2017b; Valentine and Skelton 2003), but also shared experiences of exclusion and stigma (Weeks 1996). Research links gay community to the well‐being of gay men (Handlovsky et al. 2018; Lefevor et al. 2024). As Nardi (1999) argued, gay communities were often *invincible* in their interdependence. Yet newer research shows such communities are not guaranteed (Kelly et al. 2014; Ghaziani 2016), gay men may feel ambivalent about them (Holt 2011), and there are intergenerational differences among gay men in attitudes toward ‘gay community’ (Marshall et al. 2019).

Today, Canada recognises same‐gender couples’ right to marry and co‐parent. As gay parenthood becomes more normative, some gay fathers may form families that lack interdependence with a gay community. As Gato et al. (2017) emphasise: “Social support provided by sexual‐minority peers to lesbian and gay prospective and current parents needs to be further investigated” (p. 319). They add that “the detachment from childless lesbian and gay friends” also merits investigation (p. 319). What was once essential may now be optional.

This study draws on in‐depth interviews with 18 Canadian gay fathers. It asks: (1) How do gay fathers understand their relationship to gay community? (2) Given parenting demands, what support do fathers expect and need? How do families of origin and gay community contribute? (3) If not family and gay community, whom do they rely on to raise a family? and (4) How does fatherhood change their relationship with the community?

To date, there is little research on how gay fathers negotiate their new domestic lives in relation to their gay community. To fill this gap, I analyse these mostly White, (upper‐)middle‐class Canadian gay fathers’ understandings, expectations, and experiences of and relationships with their gay community to show they describe it in terms of gay friends, ancestors, and shared ‘gay’ activities. They are grateful to past generations for securing their rights to marry and co‐parent. They desire support from family and gay friends and are determined to sustain their ties to gay community, but struggle to do so. I argue that the organisation of childcare, demands of intensive parenting, and geographic distance from other gay people, as well as their families, complicate this support–a social process I call the *gay parental turn*, in which gay fatherhood triggers a reconfiguration of ties with gay community. It implies not only the loss of friends, but a present, dynamic community reorientation in which expectations of support, shared activities, and generational connection are renegotiated. In the absence of family or past community, many fathers—especially those with older children—forge new community ties with the straight parents of their children's friends.

### 1.1 Literature Review

#### 1.1.1 Contextualizing the Privatized Nuclear Family and Families of Choice

In Canada, families are shaped by the organisation of childcare, long work hours, limited free time, geographically dispersed families, expectations of intensive parenting, and family policy. Dual‐earner households are common (Cohen and Pulkingham 2009; Dobrowolsky 2009), yet many struggle to care for children and other dependents. Outside Québec (Tremblay 2014), childcare costs often rival housing (Langford et al. 2017). While the federal government announced a $10‐a‐day childcare plan in 2024 (Prime Minister of Canada Justin Trudeau 2024), it was not in effect during these interviews. Like many other Global North countries (Newman 2012), Canada faces rising job insecurity, weaker benefits, and irregular work hours, which complicates parental leave and childcare decisions (Ornstein 2021).

Fathers in this study are highly educated dual‐earners with financial stability, yet carework remains challenging. Contemporary parenting is shaped by “intensive mothering,” which demands copious time, energy, financial investment, and the prioritisation of children's needs above all else (Hays 1996). It now extends to some fathers and across class lines (Faircloth 2023; Scheibling and Milkie 2023). To meet these demands, Luxton (2006) noted that Canadians rely on paid and unpaid caregivers—friends, neighbours, and co‐workers—but these supports are inconsistent. Provincial leave and childcare policies also provide inadequate support (Mahon and Phillips 2002), prompting calls for affordable childcare and comprehensive parental leave (Fox 2015; Michel and Mahon 2002). For gay fathers, the caregiving burden may be compounded by strained family ties and distance from gay communities—long‐standing sources of support.

In the 1980s and 90s before LGBTQ+ people won their rights to marry and co‐parent, gay and lesbian communities often relied on chosen families, blurring the line between family and community. Weston (1991) found that American gay men and lesbians developed enduring, familial ties that offered emotional and material support, including caregiving and co‐parenting (p. 207). These relationships emphasised mutual aid, love, and shared history (pp. 104–109). Though household boundaries remained, they were porous, distinguishing them from conventional, straight, nuclear families.

Queer people redefined family as an extension of friendship, inventing flexible roles that blurred relational categories (Weston 1991, p. 118). This allowed strategic naming—using “family” in queer spaces and “friend” in less accepting contexts. Also, the language of community provided a framework to locate others, share identities, and define relationships. Community was more than a setting—it was the very source from which chosen families emerged. Chosen families were not freely selected, though, as they often included others of similar race, gender, class, and age (p. 111). Community, then, shaped the renegotiation of kinship, moving away from biological ties toward chosen families (p. 18). Ex‐lovers frequently remained part of chosen families, reflecting norms of interdependence forged under homophobic conditions (p. 111).

### 1.2 Gay Fatherhood and Community

Today, scholars debate the relationship between gay men and their gay community (Armesto and Shapiro 2011; Goodfellow 2015; Forenza et al. 2021; Holt 2011). U.S. research shows gay parents in nonmetropolitan areas actively pursue gay community to cope with minority stress (Kinkler and Goldberg 2011). Goodfellow (2015) found that some U.S. gay fathers embed themselves in towns “known [to be] a centre of lesbian and gay parenting” (p. 61). Yet in heteronormative suburbia, gay fathers may unsettle expectations in spaces like playgrounds and schools. As Stacey (2006) observed, “heterosexual ‘situations’ lead most straight men to paternity, while homosexual ‘situations’ lead a majority of gay men to childlessness” (p. 27). Berkowitz and Marsiglio (2007) noted that straight men often see fatherhood as normative, while many gay men long viewed it as unattainable. Straight fathers are expected in children's spaces; gay fathers are less so. Those gay men who do become co‐fathers may struggle to integrate gay and parental identities (Schacher et al. 2005). Today's gay fathers must therefore navigate entrenched beliefs about the supposed incompatibility between gay life and domesticity.

How they do so, however, remains understudied. Research on LGBTQ+ parents suggests pressure to separate gender and sexuality from parenting (Berkowitz and Marsiglio 2007; Chabot and Ames 2004). Some gay men desire this, envisioning fatherhood through a nuclear family ideal (Rabun and Oswald 2009). Forenza et al. (2021) found that lesbian mothers and gay fathers “de‐emphasised LGB+ identities in favour of self‐categorisation as a parent” (p. 24). Armesto and Shapiro (2011) similarly found that gay fathers’ communities shifted toward other parents, with ambivalent support from gay peers (p. 89).

Also, sociologists debate how gender and sexuality shape parenthood (Underwood 2024): parenting often follows children's schedules, reflecting intensive parenting culture (Hays 1996; Scheibling and Milkie 2023). Under this pressure, LGBTQ+ identity may become secondary. Among straight couples, parenthood often means the loss of friendships and former identity (Fox 2009; McMahon and Ranson 1997). Munch et al. (1997) found that American mothers lost friendships and reduced social contact, while fathers’ networks became more kin‐centred. Jacobs and Gerson (2005) described the ‘time crunch’ of dual‐earner households with children: time becomes scarce and unequally distributed, particularly by gender and class. Yet, while straight parents face shifts in community contact and composition, they remain surrounded by other straight people and often their extended family of origin.

For gay fathers, the loss of gay community carries unique, important consequences. It may reduce their ability to endure stigma and minority stress and disrupts identity and community formation processes vital to LGBTQ+ well‐being (Russell and Fish 2016). Fatherhood may leave some gay men feeling disconnected not only from their past selves, but also from their practices of sharing gay activities and belonging to a gay community, with whom they have so long been interdependent.

## 2 Theoretical Framework

The theoretical framework for this research draws upon interrelated paradigms: (1) socialist feminist approaches to families, community, and social reproduction, (2) intersectionality and whiteness theory, and (3) homonormativity.

First, I treat family and community as analytically distinct but empirically entangled. I define ‘families’ as the social relationships formed to care for children, dependents, and adults, including friends, lovers, or ex‐lovers–a broad definition that captures the diversity of family patterns (Fox and Luxton 2014, p. 6). Yet, in this study, all couples are monogamously married and live together with their children in nuclear households, and nearly all are White, thus resembling the privatised, heteronormative, nuclear family.

I also attend to social reproduction*—*the material, mental, and emotional labour sustaining families across generations (Laslett and Brenner 1989). Key here is to ask how this is done, and who does it. This labour is compounded by intensive parenting, which demands that parents prioritise childcare above all else (Faircloth 2023; Hays 1996; Scheibling and Milkie 2023). Class further differentiates parenting capacity and resources, which are shaped by finances and free time (Lareau 2003). Despite norms of privatisation, Canadian families often rely on communities of kin and friends to meet labour needs (Luxton 2006). Together, the labour of social reproduction within entangled family and community relations raises questions about inequalities among gay men—or which men can draw support from community.

Second, to analyse these inequalities, I draw insight from intersectionality and whiteness theory (Crenshaw 1991; Doane 2003). Intersectionality examines how power shapes not only single axes of division but multiple interacting axes within the context of African American women's lives (Cho et al. 2013; Crenshaw 1991). In the context of this sample, the critical insight is that, depending on one's identities, social structures and power differentially shape life experiences and outcomes—a vital insight given that ‘gay’ and ‘male’ are broad categories (Collins and Bilge 2016). Relatedly, whiteness theory critically examines the privileges and oppressive practices of racially advantaged people—and this sample is almost all White (Andersen 2003). Doane (2003) argued that whiteness is interlocked with race, class, gender, and sexuality, implying White, middle/upper‐class, male, and heterosexual identities are its most privileged forms in contemporary Western societies.

Third, sexualities scholars argue that homonormativity sustains heteronormativity and whiteness (Duggan 2002; Ferguson 2018), suggesting certain gay identities may be normative. Yet homonormativity also describes a privatised, depoliticised gay culture grounded in domestic consumption and respectability (Duggan 2002). Thus, White, middle‐class, cisgender, and monogamous (gay) couples who co‐parent may fit within gay‐tolerant heteronormative geographies. This can exclude racialised and/or poor LGBTQ+ people, upholding norms and reproducing unequal social structures and power.

### 2.1 The Gay Parental Turn

Drawing on Anderson's (1983) view of ‘community’ as imagined among those who see themselves as part of a group, Formby (2017a) organised definitions of LGBTQ+ community into spatial, cultural, imagined, virtual, and personal connections (p. 2). She challenged the assumption that LGBTQ+ people “automatically belong to ready‐made communities” (p. 2). In her British sample, most LGBTQ+ respondents defined ‘community’ through shared values, experiences of marginalisation, and processes like ‘coming out’—in contrast with communities of “others” such as straight people (2017b, p. 168). Nardi (1999) argued that friendships and shared participation in gay activities are key to gay male communities: participation in gay bars, LGBTQ+ shops, and political organisations are important ways gay men achieve gay identity, a first step towards gay community. Others locate community in shared relationships and identification more than in geographic proximity to gay institutions (Kelly et al. 2014). Yet some gay men express ambivalence toward gay communities (Holt 2011; Wilkinson et al. 2012), while research shows that ‘gayborhoods’ are being restructured as LGBTQ+ people move into mainstream society (Ghaziani 2016)—a process closely linked to the racialised dynamics of “white flight” wherein White, middle‐class families exit urban cores for supposedly safer, family‐oriented, and institutionally resourced suburbs (Crowder 2000).

Generational belonging among gay men shapes common experiences but also exposes intracommunity tensions. Marshall et al. (2019) conceptualised ‘gay generations’ as defined by shared milestones but also highlighted how “the individual and collective experiences of [gay] generational time” are typically “out of historical sequence” (p. 571). While members of a gay generation may share milestones—like coming out, joining a gay community, or entering a same‐gender relationship—gay individuals often reach them at different life stages due to differences by social class, race and/or ethnicity, nationality and citizenship, religion, and so on. As a result, shared milestones are often “out of synch among individuals,” complicating shared identification. In this study, gay fathers marry and raise children—experiences many gay peers may not share, challenging the basis for retaining gay community (Marshall et al. 2019).

For gay fathers, what is the distinction between ‘family’ and ‘community’? Given debates on defining community, I prioritise three features to analyze the distinction: (1) identification with a shared community (friends, acquaintances, and others recognised as part of the community), and (2) participation in mutual support and gay activities (shared experiences in locations and venues that centre or serve gay people). These fathers’ location in a gay generation may also raise (3) expectations of support by gay friends. In this research, however, fathers’ identification with a gay community, participation in gay activities, and expectations of support go unfulfilled. These three features of community, then, enable an analysis of the mechanisms that cause the gay parental turn.

Parents, the demands of privatised childcare, intensive parenting, distant relatives, and gay fathers' ability to outsource social reproduction reconfigure their gay community ties. Though they retain strong identification with gay community—expressed through gratitude to “ancestors,” memories of collective struggle, and ongoing commitment to gay politics—their transition into parenthood exposes the limits of these ties as sources of material and social support. Expectations of aid go unmet, producing feelings of estrangement from a community they viewed as a site of dependable solidarity. Consequently, these mostly White, (upper‐) middle‐class fathers experience the *gay parental turn* as they reorient their community ties towards the (straight) parents of their children's peers. This shift coincides with a reweighting of identity—from “gay man” towards “parent”—and, for many of them, a geographic and social movement away from gay neighbourhoods.

## 3 Methods

The data for this article come from semi‐structured interviews with 18 fathers in Ontario, Canada: 16 self‐identified gay men, one pansexual man, and one pansexual transmasculine person. All are married and live with their children. Two couples became fathers via public adoption, one via private adoption, one (trans father and his cis husband) through natural birth and a prior relationship, and five via gestational surrogacy.

Accessing participants required years of outreach to fathers from earlier studies and queer‐serving organisations. Due to the limited number of gay fathers in Canada (Statistics Canada 2022) and the COVID‐19 pandemic, the sample size is limited; however, I continued recruitment until I reached data saturation (Aurini et al. 2022). Ultimately, participants were self‐selected, with most learning about the study through a Toronto parenting course. Others were referred by participants. I did not offer benefits for participation. All participants were assigned pseudonyms.

I conducted nine joint interviews with 18 fathers, counting each father in the sample as both voices mattered. In past work, individual interviews often repeated responses, and contradictions were rare. Fathers often needed to recall early parenting experiences, and joint interviews helped with memory recall. Time constraints made joint interviews more efficient, especially with children often present. I did not interview children, though I assisted with bottle‐feeding and bedtime.

Between October 2018 and December 2019, I interviewed 12 fathers in‐person at their homes. Due to the COVID‐19 pandemic, the remaining six were interviewed over Zoom from April 2020 to January 2021. Interviews lasted one to three hours, followed the same semi‐structured format, and varied slightly based on family composition. All began with family histories, including coming out, meeting their partner, becoming fathers, daily parenting, and their understandings of family and community.

During interviews, I shared that I was also a queer prospective parent in a gay marriage, and White and well‐educated. These common experiences helped participants feel at ease discussing personal details about their lives: several commented that I “know what it's like.” This demonstrates I am a peer but also reflects a social constructionist approach to knowledge production (Gergen 2023). It is neither possible nor desirable to separate my experiences from my analysis of these men (Aurini et al. 2022). Our shared positionalities established rapport—and therefore the quality of data I solicited—but also how I interpreted and analysed data.

All participants live in Ontario. All have post‐secondary education; four have less than a Bachelor's degree, while seven hold Master's or higher degrees (two PhDs, one MD, one joint PhD/MD). Household incomes were high and were distributed as follows: $80,000 to $99,000 (*n* = 2), $100,000 to $149,999 (*n* = 2), $150,000 to $199,999 (*n* = 4), $200,000 to $249,999 (*n* = 2), and a large plurality above $300,000 (*n* = 8).

Fathers ranged from 36 to 51 years old, with a median age of 41.5 years. Children ranged from four months to 21 years (median: 5.25). Three children were under one year, four were between one and three years old, three were between four and six years old, four were between seven and nine years old, one was 11 years old, and one was 21 years old.

12 fathers self‐identified as White/Caucasian, one as Italian, two as Jewish, one as Latino, one as Hispanic, and one as Chinese. During recruitment, I attempted to ensure diversity in race and ethnicity, but I was unable to locate more racialised fathers. The three participants who are racialised are each married to a White man. This did not allow me to generate robust analyses of differences among racial/ethnic categories. Rather, I treated the over‐representation of White participants as a finding. While not representative of the general Canadian population of gay fathers, the whiteness of this sample reflects the relative privileges required to achieve gay fatherhood in Canada today.

Data were gathered using broad, open‐ended questions, for example, “Let's talk about the day you became fathers. What was that like?” and “Do you feel more or less connected to the LGBTQ+ community today?” All interviews were recorded, transcribed, and coded using NVivo. I used memoing during and after interviews to document setting, context, and early patterns (Lempert 2007). Memos captured how social context shaped experiences of family, community, and belonging. Then, I coded line‐by‐line to generate themes (Lofland et al. 2006), focusing on how fathers met care needs, accessed parental leave, involved extended family and gay friends, engaged with and renegotiated gay (and other) communities. I compared and contrasted couples across variables—child age, geography—to identify patterns and exceptions. Finally, I reviewed and revisited interview data over several years to refine findings.

## 4 Findings

### 4.1 Before the Turn: Gay Community as Intergenerational Solidarity

In this study, gay fathers do not create ‘chosen’ families characterised by fluid boundaries and close ties with other LGBTQ+ individuals but instead resemble the privatised, nuclear model. Still, they maintain a connection to gay community, describing them as “those who came before us,” and using familial language to honour prior generations. Their accounts illustrate the preconditions of the gay parental turn, as fathers’ present family lives as anchored in histories of collective struggle and solidarity (Weston 1991).

Simon, 49 and Jewish, lives in Toronto with his Jewish husband and twin six‐year‐olds. He calls older gay male friends “uncles” and “ancestors,” crediting them with making his family possible. He says:

The rights my queer ancestors had to fight for—and some of them are my good friends and I still thank them… Thank you for giving me the ability to have kids and make these choices… It would have been completely different for us 30 years ago. I would have been in really bad shape if I couldn't make a family.

Simon's language positions “queer ancestors” as moral and political forebears. He also refers to older gay friends as “uncles.” In Ottawa, Rob and Michael describe an older couple as their “gay uncles”: “We have lunch when we're in town, but we don't see them enough.” This designation signals both affection and reverence for elders’ sacrifices and how the gay parental turn reorients them from participation in gay social environments to more commemorative, affective bonds.

Fathers frequently articulate a sense of indebtedness. Brayden, a White father in southwestern Ontario, says:

I cannot imagine my life without my kids. If I were living [here] a few decades ago, my family as it exists right now would not exist… So I know how special this is. I know how much our family owes to gay and LGBT people from the ’80s and ’90s.

This indebtedness demonstrates that Brayden understands his present family as an outcome of the collective labours of prior generations (Weeks et al. 2001), even as the gay parental turn marks his move away from gay community.

Bob, 50, White, and married to Kyle, who is Italian, recalls: “When I came out, you could not get married and have kids. That was not something gay men could do.” Of the 1990s, he adds, “We were on the streets fighting to be heard, and seen, and treated with dignity… People I knew, friends of mine, died in those years.” He emphasises community support during that time: “When I came out in the 90s, [my gay friends] were my brothers and sisters. Being with them on Church [Street] or at Pride was being with my family… my new family.” Bob's distinction between past collective struggle “on the streets” and his present family life highlights the temporal nature of the gay parental turn.

Many participants are in their 40s and 50s and came out during the HIV/AIDS crisis. The epidemic catalysed LGBTQ+ organising and legal battles (Ferguson 2018; Kelly 2011). The life chances of gay men were closely tied to the strength of their communities. Bob states, “I would not be where I am today without my community. I would not be a father, married to Kyle, and living a life I didn't even know was possible 30 years ago.” Simon adds: “Where would I be today—where would we all be today, if not for those gay men that died… and survived to fight to ensure this never happened again?” These generational narratives situate the gay parental turn: prior experiences of community as a site of survival and care (Nardi 1999) give way to a present in which parenthood reorganises their activities and relationships.

These fathers recognise that marriage and parenthood were made possible through collective struggle. Their comments reflect a historically interdependent relationship between gay men and the broader LGBTQ+ community. They describe their community using familial terms—“uncles,” “ancestors,” “brothers,” and “sisters.” To them, LGBTQ+ communities are the very foundation upon which their current family lives rest.

### 4.2 Driving the Turn: The Reorganization and Privatization of Care

Many fathers maintain relationships with extended family, a key support for new parents (Fox 2009). It was the first domain I examined when exploring how fathers care for their children. Four patterns emerged: (1) two fathers rely on a grandmother's help, (2) four alternate work and home time, (3) eight keep the higher earner at work, and (4) four share leave and then hire or plan to hire childcare. These arrangements illustrate the social and material mechanisms for the gay parental turn: childcare is organised primarily within privatised families (Fox 2009; Luxton 2006).

In one early interview, I drove several hours to meet Steve and Javier, who lived with their two‐year‐old and four‐month‐old twins. Steve's mother, who greeted me while unloading the children from a minivan, had watched them that afternoon. In the kitchen, she warmed bottles while we sat on the sofa with the babies and toddler. Steve, a White man, says, “We could not be luckier. Mom lives nearby and helps out with the children all the time. She's retired.” Their case is an exception that clarifies the pattern. When family support is available, the pressure driving the gay parental turn may be softened, yet this experience is unique in this sample.

Steve took “some time off” with their first son, and Javier, his Latino husband, took two months, but Steve's mother “dominantly took care of [their firstborn]” and now helps with the twins. Javier says he “would have liked to take more time off” but couldn't afford it. Both had also taken time off work pre‐birth to visit and support their surrogates in Latin America. They discussed hiring a “Spanish nanny” with Steve's sister, but “because of financial reasons,” it was not possible.

This portrait of two gay fathers supported by a grandmother fit the vision I expected while designing this research project. Yet the rest of the sample revealed more complexity. In the eight other households, no father reported consistent family help. Some received short‐term support from visiting relatives, but most lived far away. The lack of family support intensifies the gay parental turn by concentrating social reproduction within couples, limiting the time and energy available for sustaining gay community ties.

Some fathers alternated work and childcare. When I visited Carl and Viggo, both White, in Toronto, Viggo's exhaustion was obvious. They had recently adopted a 4.5‐year‐old, and Viggo said, “We have no one!” Aside from brief help from the child's foster mothers, they were on their own. Neither took parental leave. With dark circles under his eyes and regular yawns, Viggo explains:

We're able and willing to rearrange our lives and finances to make it work. And, parental leave, the way it works, it's a better deal in a sense for lower income families because […] for us, it was a huge drop in income.

He found leave “options lacking,” and says “the cost of childcare is a huge burden.” Though they could afford it, they chose to reorganise their finances instead. Parental leave would reduce their income, and childcare costs averaged $12,800/year per child in Ontario in 2018 (Jamasi et al. 2019). They had not hired a nanny or babysitter. Both worked long days, alternating so one was always home. They wished for family help, but both sets of grandparents lived several hours away by plane. This fatigue and time crunch (Jacobs and Gerson 2005) reveals how the gay parental turn is a process that erodes their capacity to participate in activities other than parenthood—and certainly complicates participation within the rhythms of gay community life.

White, trans father Michael took a year of Employment Insurance [EI] leave after each birth while his White husband, Rob, worked. When Michael returned to work, they did not hire help, instead alternating schedules to ensure one parent was always home.

The largest group sent the higher earner back to work soon after birth. White, adoptive father Teddy took three months’ leave: two weeks’ vacation and 10 weeks of [EI] parental leave. His Hispanic husband, Juan took six months. Their adoption agreement required they take nine months total, and since Teddy earned more, Juan stayed home longer. With Teddy's family in the U.S. and Juan's in Latin America, they had no family help, and they did not hire caregivers. Teddy notes that though his Christian parents visited after the adoption, their support was limited:

My parents came up from Michigan […] after we got him. They stayed for a few weeks and we did touristy things. They had a hotel nearby but we got to spend a good amount of time together when [our son] was just settling in.

When I asked whether they helped at home, he said, “No, not really. We went out for food and around the town. They weren't very enthusiastic about coming into our home [*laughs*].” Their relationship had been strained since he came out 20 years prior. Happily, the arrival of a child offered a limited opportunity to reconnect. Here, the gay parental turn is deepened by homophobic family relationships, as tenuous ties with the family of origin provide little support, displacing the need for support towards community.

Simon and Shlomo were similarly unsupported. Their surrogate took 17 weeks’ leave but did not care for the babies. Shlomo used the remaining 35 weeks of [EI] leave and added 10 weeks of vacation. He remarked, “Since becoming dads, we have had an insufficient social welfare net!” and noted, “We're pretty isolated because we're not from Toronto. I'm from [rural Ontario] and Simon is from Michigan, so all the support we might have gotten from family, we didn't have.” He added, “It was sad that our siblings didn't want to help out too,” even those nearby. With limited family help and disinterest from gay friends (described below), they relied on their high income ($150,000–200,000CAD), hiring babysitters and a cleaner. Purchasing care reveals how class privilege reshapes but does not eliminate the gay parental turn. Outsourced care may substitute for family and community but offers no relational depth.

Austin (White) stayed home nine months with each infant, but couldn't take a full year because he hadn't given birth. His husband saw this as a system flaw: “It should be twelve months!” Occasionally, they hire a teenager to babysit: “just a couple hours here and there… so we can go out to get stuff done.” Their income (over $300,000CAD) allows them to buy help when needed. Even here, outsourced care is a stopgap that gets the job done, but at the cost of community ties.

Anthony (White) took two years off after his twins were born. His husband, Dan, a Chinese physician, added six weeks of paid leave. Anthony took one year of paid leave and another year of unpaid leave to stay home with the babies. They hired a nanny after returning to work, but it did not work out, so the twins were placed in daycare.

Four fathers shared leave, then hired or planned to hire childcare. Kyle took six months’ [EI] leave; Bob took four. During those ten months, Kyle recalls “a few times where we had appointments, and we dropped our son [off] at his grandparents’, but not often.” They considered a nanny, but by 14 months, their child entered childcare until kindergarten.

René and his husband, both White fathers, took two months off after their infant's birth. When I interviewed them, their baby was four months old, and they planned to hire a nanny. Both of their families lived in the U.S., so it did not help.

Across cases, parental leave and outsourced care reflect how institutions—EI rules, workplace policies, childcare markets (Cohen and Pulkingham 2009)—structure the gay parental turn, organising life around parental and economic obligations, rather than community immersion. Yet fathers managed work and parenting differently. Though high incomes enabled some to buy support, most found the costs burdensome and were left more on their own than expected. Aside from Steve's mother, few received consistent family support. This unmet need primes them to expect support from gay community, given long histories of interdependence—only to encounter the limits of those ties under conditions of privatised, intensive parenthood.

### 4.3 Turning Towards Gay Community, Then Turning Away

Gay fathers in this study typically came out in adolescence or early adulthood and sought others like them in LGBTQ+ venues. At this stage, the LGBTQ+ community offered exposure to queer culture, social opportunities, and a sense of identity and belonging distinct from their upbringing (Formby 2017a; 2017b; Nardi 1999). Many turned to these communities for support and connection. These past experiences shape the gay parental turn as community is remembered as an infrastructure of belonging and mutual support.

Viggo hopes to maintain ties with other gay people as a parent. He and husband Carl pledged to stay connected with their “old life.” Viggo says, “We hoped we would have some of our [gay] friends over to play with our son, help around the house, you know, but no one showed interest in that.” His comment reflects broader patterns in these interviews: these fathers want to parent in queer community and expect both emotional and practical support. They invest effort in maintaining friendships and feel disappointed—and often abandoned—when support does not appear. Here, the gay parental turn captures a clash between historical interdependence and the present reorganisation of their lives towards parenthood.

#### 4.3.1 Fathers’ Desire to Parent in Community and Expectations of Support

Most respondents want to parent within LGBTQ+ communities. Steve, 41, father of three, says, “I would love my children to be around a rainbow of adults… lesbians, gay men, trans people, people of all races and abilities… That would be my ideal situation.” For many fathers, raising children among queer peers is ideal. René, 40, in eastern Ontario, says, “I want my child to know the gay community… I am proud of who we are… to share our community with my kid, and future kids.”

In a small southern Ontario city, Brayden and Austin find fewer public LGBTQ+ events, making it harder to build queer community. Brayden notes, “It sure would be nice to have other gay parents around… but it's hard.” Most fathers live in (heteronormative) suburbs, which complicates shared activities with other gay people (Ghaziani 2016).

In Toronto, Anthony and Dan made efforts to keep gay friends involved after becoming fathers. Asked what he hoped from gatherings with gay friends, Anthony said: “Well, some show of support! Offers to babysit, to help with dishes, laundry, that sort of thing.” He underscores expectations of emotional and practical support—gestures of friendship, childcare, and domestic help, or key features of social reproduction (Laslett and Brenner 1989).

Teddy and Juan, Carl and Viggo, Bob and Kyle all echo this. Juan says:

At home [in Latin America], my grandmother took care of us kids, but we have no grandmother here. We want to have good relationships with our gay friends so they can help take care of him, to babysit sometimes, to help us around the house… but it's just us.

These fathers’ expectations reflect longstanding traditions of mutual support among gay men. Yet even as they seek to maintain these ties while parenting, their efforts often falter. The gay parental turn is thus driven not by lack of desire for community, but by a mismatch between fathers’ care obligations and the norms of their gay community.

#### 4.3.2 Fathers’ Efforts to Sustain Relationships With Community

Fathers in this study are determined to stay connected to community. Viggo and Carl missed nights out in the Church‐Wellesley neighbourhood and organised ‘daddy date nights’, but their son struggled with a babysitter. They invited friends over instead, but plans fell through. Frustrated, Viggo exclaims, “I don't feel gay anymore!” capturing how the gay parental turn limits participation in shared gay activities and thus in gay community.

Like other fathers, Viggo associates “being gay” with participating in gay social life. Parenthood disrupts this, making it harder to “feel” gay. Teddy echoes this sentiment: “It's hard to feel gay when you can't find [other gay people] anymore.” As Carl recalls, they tried to hold on to that sense of belonging. Shortly after becoming fathers, Carl travelled to New York for work:

I went to New York. I was hesitant because we were in this high stress period [of parenting]… I am so thankful I went because […] I was gay again, and I went to a theatre show. That's right! I went with my friend to a gay bar and we had cocktails and, you know, [*laughs*] ‘Bring on the penises! Let's be gay! Hallelujah!’

He emphasises how vital it was to keep his ‘gayness’ alive: “I mean, we're monogamous, but just to be around so many other gay men was nice.” Speaking to Viggo, he adds, “Actually, it was my fault that you didn't get your gay day after I got back.” For them and others, friendships are central to sustaining a sense of gay community. Still, most fathers report that such connections are hard to maintain. During the gay parental turn, gay community experiences become episodic.

In Toronto, Anthony arranged visits from gay friends after becoming a father to twin boys:

We would have gatherings of our gay friends […] a first visit to meet the boys, and we could tell pretty quick who was interested… Some friends who didn't want to have kids came around for a while, but eventually it petered out… Invitations to go to others’ places started to disappear…

Although these gatherings faded, they remained meaningful. Many fathers built connections with other LGBTQ+ parents while preparing for fatherhood. Several attended a gay parenting course where they met other prospective fathers. Teddy, who took the course, tried to maintain the group: “I got everyone together on Facebook and tried to get us together a few times, but I guess the interest just fell off.” His husband, Juan emphasised their son's need for community: “I wanted [our son] to meet other gay parents […] because it is important that he felt a part of [the gay community].” Despite setbacks, they continued attending LGBTQ+ family events like Toronto Pride. These fathers demonstrate great efforts to build family‐centred gay activities shared with gay community, but to little avail, highlighting the transformational power of the gay parental turn.

Brayden and Austin also pursue friendships with other gay people. They bring their two children to Pride. Asked why they attend, Brayden says, “Well, we want gay friends! It would be great if they had kids too and could, you know, could come around the house and hang out, help out.” Whether through hosting friends, going out, building ties with other queer parents, or attending Pride, these fathers strive to stay connected to gay community—often without success. Their persistence shows that the gay parental turn is a constraint, not a choice.

#### 4.3.3 Fathers’ Sense of Abandonment by Community

While fathers strive to maintain ties with their gay community, many express disappointment and a sense of abandonment. Juan hoped gay friends would help care for their son, but admitted, “it's just us.” Viggo echoed this: “We don't have family around us, and our gay friends are [away without leave], basically, so it's just the two of us.” These accounts highlight the negative consequences of the gay parental turn at a moment of great need, compounding the demands of intensive parenting (Hays 1996).

Brayden, who brings his children to Pride in Toronto, was disappointed by the family‐friendly offerings:

[The LGBTQ+ family events at Toronto Pride were] like a sad joke! There were almost no families there, but lots of single people who wanted to have families. I would love to see more things that connect gay families out there that are positive, but we have not found those yet.

Asked for examples, he replied, “Events appropriate for children and parents, you know, bouncy castles, cotton candy, other kids playing and parents talking…” Although Pride includes family spaces, every father in this study critiques them. Teddy recalled:

“We took [our son] to the family area, but we didn't last long. [Our son] got bored quickly. There wasn't much to do… there were not many parents and children. Everything interesting was happening somewhere else.”

These criticisms reveal an institutional gap. Gay community is little organised around family life, reinforcing the structure of the gay parental turn towards heteronormativity.

In Toronto, as Carl and Viggo reflect on helping each other ‘be gay’ while juggling work and parenting, they acknowledge a gap. Viggo, comparing himself to Carl's trip to New York, shares disappointment with his local gay circle:

There are a lot of friends that we haven't seen because they [have] no interest in children, even like the children in their families. It's not just our child; it's children they don't like. So, we haven't seen them in two and a half years.

Some fathers were explicit about feeling abandoned by their community. Simon described becoming a gay father as being dumped by friends: “Our friends came over and saw the kids one time and then dumped us! We were kind of like weirdos basically for getting married and having kids, so that wasn't a real highlight….”

This surprised me. I had assumed gay fathers would still feel like ‘weirdos’ in straight, cisgender spaces, not among other gay men. Yet in parenthood, these fathers felt less strange among other parents (mostly straight and cis), and more so among gay friends. This reversal highlights the significance of the gay parental turn. Unlike straight parents who remain within the dominant sexual community, gay fathers move into social worlds where their sexual minority status diminishes relative to their parental status (Schacher et al. 2005).

Simon described political tensions with his friends:

I had some friends who labelled me an assimilationist and were offended that I wanted to get married and have kids… I said to one of them ‘I'm a little bit bothered that you're having such a strong reaction to my getting married and having kids… but you should know I'll be there fighting for your rights to be who you want to be’. It felt like we had to perform our queerness in certain ways. We don't want to go out to bars and we're too tired to go out to protests most of the time.

His experience reflects a common theme in parenthood—queer or straight: it leaves little time or energy (Fox 2009; Jacobs and Gerson 2005). His friends seemed more alienated by his parenting than his marriage, perhaps because fatherhood disrupts typical gay activities. As discussed, many fathers experience ‘being gay’ through shared activities. For Simon and Shlomo, this included protest—an important rite of passage and social practice that becomes challenging after becoming parents. These men suggest that gay family life might feel more communal if gay friends participated in childcare and domestic tasks.

Fatherhood also strained Bob's social ties. He recalled:

We were very involved in sports, culture, and the bar scene to some degree when we were in our twenties, and most of our [friends] were gay…. That really changed in the first years after [our son] came home. Our social circle shrunk a great deal. We had less time to engage with folks… our friends weren't very supportive, weren't interested in socialisation that involved someone who might pee or poo on them. […] Some of our friends pulled away quite intentionally.

Losing these connections left Bob and his husband without the social resources they had counted on. He also noted they “had less time to engage with folks.” Parenthood and full‐time work produce the “time crunch” or work‐family divide (Jacobs and Gerson 2005), as time once spent socially shifts to children and chores. Like Shlomo and Simon, and Bob and Kyle, Carl and Viggo also felt this strain when they took turns to ‘go out’ and ‘be gay.’ Thus, the gay parental turn involves not only a loss of community but, as I show below, a reorganisation of where care, recognition, and belonging are found in gay fathers’ worlds.

### 4.4 The Case of Rob and Michael: Sustaining Gay Community When There is No Choice

Unlike others in this study, Rob and Michael, both White, became visibly queer after having children. For them, parenthood deepened their need for gay community. Rob, a 39‐year‐old cis man, and Michael, a 41‐year‐old transmasculine person, were intentional about raising their children within an LGBTQ+ community. This reveals the limits of the gay parental turn: when homonormative respectability is unattainable, opting out of gay community is not viable.

Rob and Michael were once perceived as a straight couple. After Michael came out as transmasculine, their lives became visibly queerer. One child is Rob's biological son from a previous relationship; Michael carried their other two children early in his gender transition. Though he had been out as bisexual, Michael notes, “I was never really immersed in queer culture.” During pregnancy in his 30s, amid heightened gender dysphoria, he asked: “Where are the people like me? I want my children to know we are not the only ones, the only family that is like this.” Their intensified need for community suggests that gender history and embodiment also shape the gay parental turn—without homo‐ and cis‐ normativity, it is difficult to opt out of gay community.

As a pregnant man, Michael experienced growing anxiety: “My anxiety was increasing in social situations, so I began to stay inside […] and Rob would take on more of the activities outside the home.” Eventually, isolation prompted him to seek out queer community: “I couldn't stay inside forever. I needed to be out with my people, to just exist as myself without feeling like I stood out.”

They joined groups to meet other queer dads and find support—babysitting, playdates, and outings with other trans and gay parents. Some efforts succeeded. Michael shares: “we swap playdates and babysitting with [queer fathers].” This is social reproduction in community, distributing childcare among many families, rather than privatising it within the couple.

While many hoped to maintain gay friendships, only Rob and Michael succeeded. They also faced greater minority stress as transgender fatherhood can magnify experiences of transphobia, homophobia, and alienation (Rotondi et al. 2012). Their story suggests that ties to gay community may be sustained when they are essential, as they were for Michael's mental health and ability to navigate public life as a pregnant man with a male partner. It also reveals that homonormativity and cisnormativity are integral to the gay parental turn, as their inability to fit in with straight, cis people anchors them to queer community rather than turning them away from it (Duggan 2002).

### 4.5 A New Community? Befriending the (Straight) Parents of Their Kids’ Friends

Historically, LGBTQ+ parents struggled to build friendships with straight, cisgender parents due to stigma, particularly around gay men and children. Many felt compelled to live near other LGBTQ+ people. In this study, gay fathers have weaker ties to gay communities, but most do find community—just not where they expected. This shift illustrates the later phase of the gay parental turn. As caregiving demands intensify, fathers reorient towards reliable sites of social reproductive labour; ones structured by heteronormative and White middle‐class norms.

Newer parents like Carl and Viggo, Max and René, Teddy and Juan, and Steve and Javier report early isolation (though Steve and Javier had help from Steve's mother). Fathers with older children, such as Bob and Kyle, Anthony and Dan, and Rob and Michael, describe a new, growing community. They find it through their children—at school, in sports, or around the neighbourhood—when their children befriend peers whose parents become adult contacts. These friendships sometimes lead to playdate swaps or shared babysitting, offering much‐needed relief. Here, community is formed not around fathers’ sexual identities, but around children's lives.

Some fathers seek gay community for themselves; others hope to build one for their children. Aware that their children may be the only ones with two dads in their class, fathers reflect on how to protect them from exclusion and help them feel they belong. I asked participants how important it was for their children to be part of a gay community. Bob and Kyle say they asked their 11‐year‐old, “Is it important to you to be around other kids who have gay parents? Who have two dads or two moms? Who have been adopted?” They say their 11‐year‐old responded that it's part of his history and his identity, but it is not significant to him yet. At this moment in the gay parental turn, gay community becomes heritage rather than an infrastructure of support and belonging. In the new heteronormative community, gay community goes from a necessary source of interdependence to a symbolic, past resource.

Several fathers let their children lead in forming connections. Kyle elaborated: “It's up to [our son]!” This deference reflects intensive parenting, which prioritises children's preferences and needs (Hays 1996). It also complements homonormative ideals of “good” parenting, where responsible parenting is attained through investment in children's development, rather than in sustaining gay community ties.

Many fathers report gay friendships gradually replaced by straight ones. Bob reflects:

We became quite intentional in developing our friendships with other parents… all those parents are straight! So, our gay network was shrinking quite a bit and our straight network began to grow. And we spent more than half our socialisation time with the parents of our son's friends, and a lot less with our older gay friends…

He acknowledged the shift in his “social network” (a term he equates with friendships), noting that while he once depended on LGBTQ+ friendships for support, the change feels less significant now. “There is a sense of loss, but not of huge significance. We still have our gay volleyball team, at least until Covid hit.” The team still offers a link to gay life—though not one connected to their role as gay fathers. Bob's reflections differ from those of newer fathers like Carl and Viggo, Teddy and Juan, and Shlomo and Simon, who are still navigating early parenthood and shifting community. Bob seems to have adjusted to life in a suburban, mostly straight community, reflecting how whiteness, couple privilege, and middle‐class status can easily enter heteronormative parenting worlds (Doane 2003).

Shlomo and Simon also report that their efforts to maintain gay community have given way to friendships with straight parents. Shlomo recalled: “We met some other gay parents and hoped to become friends and swap babysitting, but you know, it's like, ‘oh just because they are gay and have children too, it's not like we're going to be fast friends or new friends, right?” Simon adds:

Ultimately, it's the children […] dictating who we hang around with, right? … I know when I grew up I wanted to hang around kids of my parents’ friends, but as the parent, you also want to hang around the parents of your kids’ friends.

Letting children “dictate” social connections reflects intensive parenting norms. While they sought support from a gay community, many reorient their social lives around their kids when those efforts falter. Here, the gay parental turn is complete: rather than building collective gay infrastructures of care, these fathers plug into pre‐existing, heteronormative geographies organised around schools and suburban neighbourhoods.

Brayden similarly finds his community becoming straighter. Living in a conservative region, he long relied on trips to big cities for gay connections. Asked if he wished he had a gay parenting community nearby, he replies:

We prefer to hang out with people that we like, and we don't really care if that means they are gay or straight… and we don't want our kids to care who we are hanging out with either, just that we want to hang out with people that we like.

As aforementioned, Brayden said it “would be nice” to have gay parents nearby. Ultimately, however, he values compatibility above shared identity. His finances and distance from urban gay neighbourhoods allow him to opt out of community. Asked whether shared identity mattered in building relationships—dimensions social scientists once saw as shaping LGBTQ+ families of choice (Weston 1991; Weeks et al. 2001)—Brayden said they should not. This view contrasts with most fathers here, who expressed a preference for LGBTQ+ friendships. These identity‐neutral or colour‐blind framings also reflect whiteness, where social comfort is mistaken for universality, and the inequalities of belonging go unnoticed.

For fathers with older children, these new straight‐parent ties bring both companionship and practical support. Bob shared: “We would send [our son] over to their places for playdates when he was younger, and they would send their kids over to ours… It gave us time to get things done or just… relax.”

Anthony and Dan also benefit from this exchange. Dan said: “Whenever Anthony and I need a night off, we call [our friends] and ask if they can take the boys, which they often do. And we do it for them too. So, it's a give and take, a win‐win for all of us.” These neighbourhood connections, rooted in their children's friendships, enable breaks from parenting.

Those with older children report new friendships and support. Like most parents, they face time pressures, work‐family struggles, and fatigue. Yet their privileges allow them to live far from urban LGBTQ+ neighbourhoods, where families of choice often formed out of necessity. These fathers build new communities where their children presently live—spaces of least resistance shaped not by identity, but by convenience, parenting needs, and available support. Here, an intersectional lens highlights the uneven nature of the gay parental turn. Racialised, lower‐income, or gender nonconforming gay fathers likely encounter greater barriers in the same spaces, revealing that the turn away from gay community is not only a constraint, but paradoxically, a class, (cis)gender, and race privilege—and a strong parallel to the sociological process of white flight (Crowder 2000).

## 5 Discussion

This study explores mostly White, (upper‐) middle‐class Canadian gay fathers’ expectations and experiences of parenthood, family, and community two decades after legal recognition of same‐gender families. Fathers varied in their approaches to parental leave and childcare, seeking support from both families of origin and their gay community. Distant relatives and strained family ties due to homophobia heightened expectations of support from gay community—expectations that went unmet. Fathers described feeling abandoned by former gay friends who visited once or twice but did not return, despite efforts to sustain those ties. Especially among those with older children, many turned to straight parents—often the parents of their children's friends—for connection and support. These patterns reflect the *gay parental turn*—the process by which entering intensive gay fatherhood triggers a reconfiguration of community ties. Fathers’ previously taken‐for‐granted relationships, expectations of care, and community support are diminished and renegotiated. With greater class, racial, and gender privilege, many live apart from gay communities they feel have rejected them—a choice not available to all, like Rob and Michael. The gay parental turn thus captures not only the loss of former friendships but a broader reorganisation of community ties in response to the demands and responsibilities of primary fatherhood.

These findings deepen sociological debates across four key areas. First, as with straight parents (Fox 2009; McMahon and Ranson 1997), gay men's identities shift dramatically after becoming fathers. Previously immersed in “gay” activities—bars, theatre, friendships—these men face the demands of full‐time work and intensive parenting (Hays 1996; Scheibling and Milkie 2023), which reduce time and energy for maintaining social ties. Their gay identities give way to parenthood. Yet unlike straight parents who remain embedded within communities of other straight people, the gay parental turn represents a significant sociological break.

Second, their diminishing friendships mirror straight parents’ experiences. In Canada and the U.S., parents increasingly organise their social lives around their children's needs and schedules, reflecting intensive parenting (Hays 1996; Scheibling and Milkie 2023). As with other parents, these men prioritise caregiving over personal ambitions, which weakens their external social relationships (Munch et al. 1997). Many call on caregiving communities (Luxton 2006) but find them unavailable. The gay parental turn thus illuminates the contingent nature of gay community: friendships and mutual support that were once reliable begin to disappear—at least for those fathers who can do without. Their racial and (cis)gender privileges, and their financial resources, buffer them somewhat— some can purchase childcare or outsource domestic labour—but this does not fully protect them from the burdens of privatised care. Some cannot afford childcare, while others trade shifts with partners or work long days to have time off later. The gay parental turn shows that whiteness, (cis)gender and class privilege can mitigate but not fully resolve the relational and social gaps this transition creates.

Third, social class shapes their experiences of privatized care. While most are stably employed, labour market precarity and weak social support heighten pressures. Fathers report wide variation in how they take parental leave—from EI, employer benefits, or vacation days—highlighting a fractured and inequitable leave patchwork. Often, the higher‐earning partner returns to work first. This research supports prior calls (Tremblay 2014) to provide Canadian families with predictable, uniform and longer‐term parental leave that allows them to transition themselves and their infants and adopted children into family life. Notably, these structural inequalities imply differential experiences in the gay parental turn: gay fathers with greater economic resources and racial privilege are better positioned to turn towards straight community in a heteronormative geography.

Fourth, the gay parental turn also lends further insight into debates about how LGBTQ+ community is reorganised within racialised and classed geographies. While there is conflicting evidence whether gay fathers locate themselves among other gay and lesbian parents (Armesto and Shapiro 2011; Goodfellow 2015), these Canadian gay fathers do not do so. Like white flight (Crowder 2000), the gay parental turn is experienced as child‐focused and practical—about support, schools, and affordability—but it has social and material consequences. It redistributes capital away from historically gay neighbourhoods and towards heteronormative institutions that already concentrate resources (Ghaziani 2016). It is less clear how gay fathers with fewer privileges navigate this transition.

This analysis is limited to 18 Ontario‐based, mostly White, financially secure, and well‐educated gay fathers. Much remains unknown about those experiencing greater marginalisation. How do low‐income and racialised gay father couples navigate parenthood and community? What about those in rural areas with little LGBTQ+ community? How do gay fathers without the option to “opt out” of gay communities build support?

Despite these limitations, this study provides insight into how some gay families are forming amid Canada's expanding legal and social recognition of LGBTQ+ rights. With access to marriage and parenthood, gay men now build families under conditions like their straight, cis peers—at least those with comparable resources. The gay fathers in this study, especially those with greater means, form families not out of necessity or resistance, but instead by richer access to conventions of family legitimacy—ones available thanks to the generations of gay ‘ancestors’ that came before.

## Conflicts of Interest

There are no conflicts of interest in this research.
